# Lignans: a versatile source of anticancer drugs

**DOI:** 10.1186/s43088-022-00256-6

**Published:** 2022-06-04

**Authors:** Minky Mukhija, Bhuwan Chandra Joshi, Partha Sarathi Bairy, Anurag Bhargava, Archana N. Sah

**Affiliations:** 1 Ch. Devi Lal College of Pharmacy, Buria Road, Bhagwangarh, Jagadhri, 135003 India; 2 Department of Pharmaceutical Sciences, Faculty of Technology, Kumaun University, Bhimtal Campus, Nainital, Uttarakhand 263136 India; 3 School of Pharmacy, Graphic Era Hill University, Clement Town, Dehradun, Uttarakhand 248001 India

**Keywords:** Lignan, Anticancer plants, Podophyllotoxin, Cytotoxicity

## Abstract

**Background:**

Cancer is considered as the second deadliest disease globally. Plants have continuously offered unique secondary metabolites with remarkable biological applications. Lignans have gained great importance due to their biological activity. Previous studies revealed that the most remarkable bioactivity of lignan class of molecules is anticancer. They are derived from the oxidative dimerization of two phenylpropanoid units. This review covers the isolated anticancer lignans and their mechanistic aspects.

**Main body:**

A bibliographic investigation was performed by analyzing the information available on anticancer lignans in the internationally accepted scientific databases including Web of Science, SciFinder, PubMed, Scopus, and Google Scholar. In this review we have tried to sum up the isolated anticancerous lignan, its source, active plant part, extract and various cell lines used to establish different studies. Here we have included a total number of 113 natural lignans. Many studies that mainly performed in human cell lines have reported. Very few plants have been evaluated for their in vivo anticancer activity.

**Conclusion:**

It can be concluded that in near future the lignans may be an effective pharmacon for the treatment of cancer. Fruitful areas of future research may be in modifying natural lignans or synthesizing new lignans with structural diversity and potent pharmacological activities. Extensive studies are needed to be done highlighting the mechanism of anticancer action of explored and unexplored plants. The data will definitely attract many researchers to start further experimentation that might lead to the drugs for the cancer treatment.

**Graphical Abstract:**

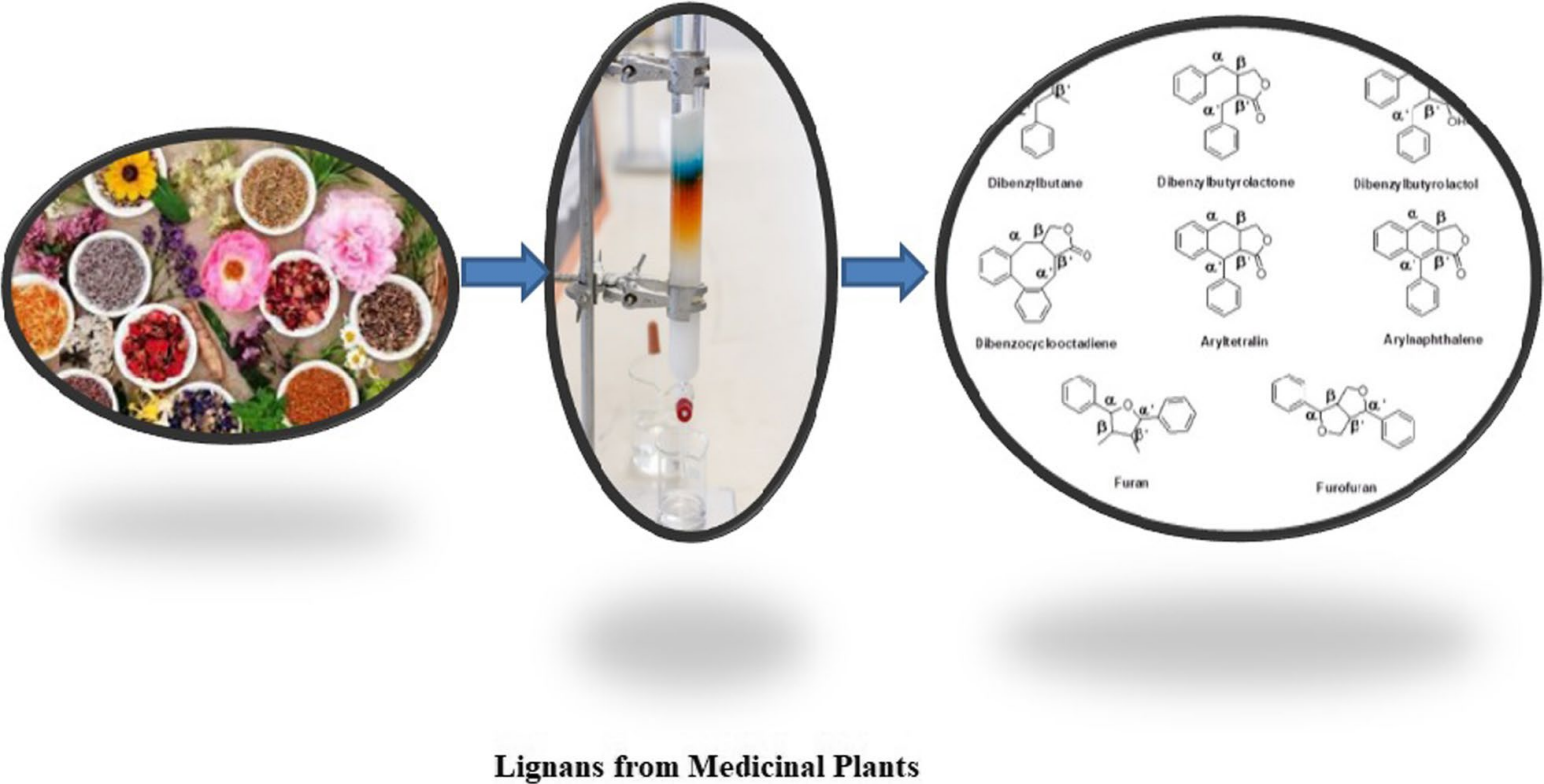

## Background

There is a great burden of disease internationally and cancer is in the top priority due to its high incidence rate that causes disability and premature mortality among human populations [1].

Cancer is not a single disease but it is a group of 100 different and distinguishing disorders that affect the entire physiological balances [2]. It is an uncontrolled growth of cells that have damaged DNA expression [3]. If the spread of these abnormal cells is not managed with certain means, it can lead to worse situations or may be death. These abnormal cells are termed as cancer cells, malignant cells, or tumor cells. Many cancers that comprises of abnormal cells are further recognized by the name of the organ that the abnormal cells originated from (for example, breast cancer, lung cancer, prostate cancer, and colorectal cancer). There are various kinds of cancers depends upon the type of genes associated with specific cancer like sarcomas, carcinomas, leukemia, and lymphomas. Carcinogenesis is a multi-leveled process consists of three noticeable stages, i.e., initiation, promotion, and progression [4]. It is the prime result of disturbances that occurred in two types of genes, tumor suppressor genes (TSG) and oncogenes.

**Fig. 1:**
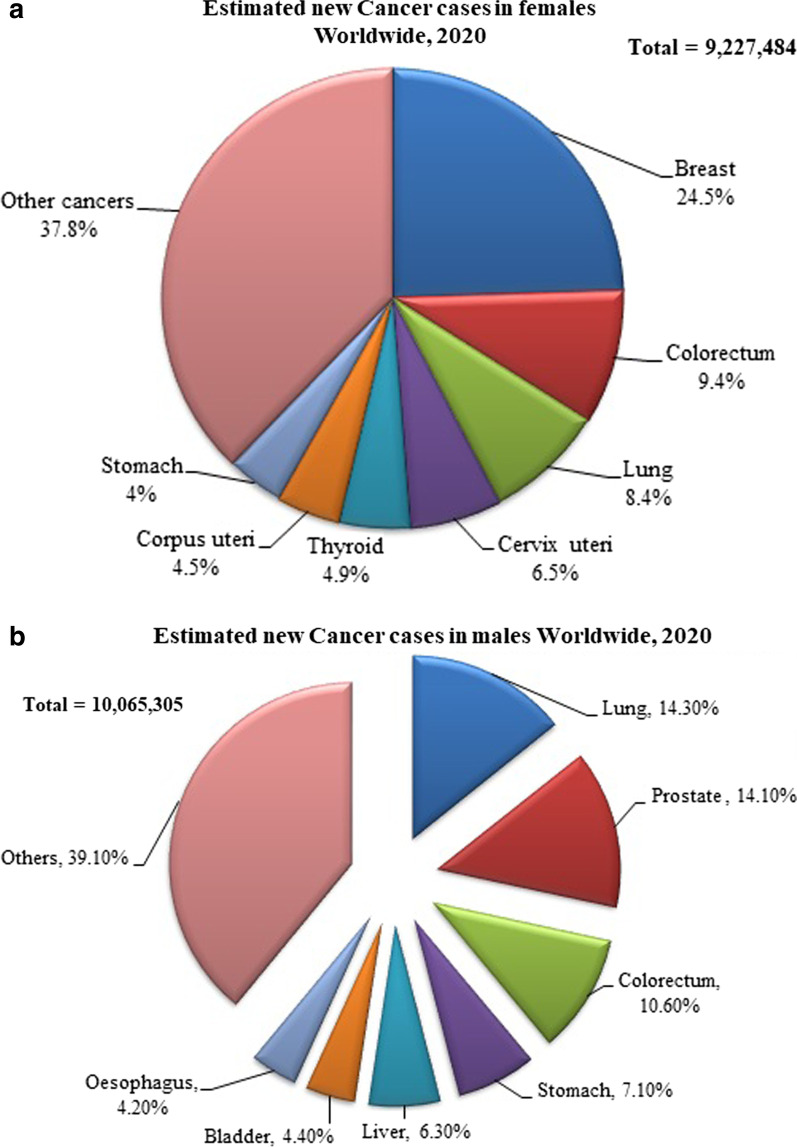
**a** Global cancer cases distribution types including all age groups of females. Source: GLOBOCAN, 2020. **b** Global cancer cases distribution types including all age groups of males. Source: GLOBOCAN, 2020

Deaths from cancer are rising continuously worldwide with an estimated 11.5 million deaths in 2030 [5]. The International Agency for Research on Cancer (IARC) estimated a shocking number of 19.3 million new cases including every possible distribution criteria (Fig. 1a, b) and approx 10 million of reported death worldwide [6].

Globally, non-communicable diseases (NCDs) accounted for 71% of total deaths. In India, NCDs were estimated to account for 63% of all deaths, and cancer was one of the leading causes (9%). The projected number of patients with cancer in India is 1,392,179 for the year 2020, and the common five leading origins are breast, lung, mouth, cervix uteri, and tongue [7]. Persons with any type of existing cancer are prone to get affected with coronavirus (SARS-CoV-2), and it is a deadly combination for individuals [8]. Studies revealed that prostate and breast cancer constitutes major types of cancer found, respectively, in men and women [9]. In children the blood cancer and the cancers related to the brain and lymph nodes are more frequent than other types of cancer [10, 11]. There are certain risk factors that increase the development of cancer in any person such as ageing, tobacco, ionizing radiation, some chemical compounds, some viruses and bacteria, alcohol consumption, family history of cancer, certain hormones, and overweight [12].

The treatment options of cancer involve surgery of tumor, radiotherapy and chemotherapy depends upon the stage and location of tumor [13]. But these treatments are very costly and require highly specialized health professionals [14]. Additionally, these chemotherapeutic agents are not free from side effects like myelosuppression, mucositis, alopecia, cardiotoxicity, neurotoxicity, immunosuppression, etc. An ideal anticancer drug would specifically be cytotoxic toward the cancer cells only and research findings suggests that phytochemicals and their derivatives are emerging alternatives for better and less toxic chemotherapeutic agents [13].

Various active compounds such as podophyllotoxin, vincristine, vinblastine, taxol, etc., have been isolated from plants, and these molecules acted as lead metabolites to modify and yield analogues better than the parent compound for activity with low toxicity and improved bioavailability [15–17].

There are diverse classes of secondary metabolites which are biosynthesized by plants and, among them, lignans are identified as the major group of natural products with a broad range of important bioactivities.

## Main text

Lignans are the class of plant secondary metabolites derived from the phenylpropanoid pathway and was first introduced by Haworth [18]. They play an important role in plant protection and are also proved to be fruitful in human nutrition and medicine [19]. The chief sources of dietary lignans are various vegetables and fruits, legumes, whole grain cereals, and oilseeds [20, 21]. Sesame and flax seeds are the edible plant components which are the most concentrated sources of lignans [22].

### Chemistry of lignans

**Fig. 2:**
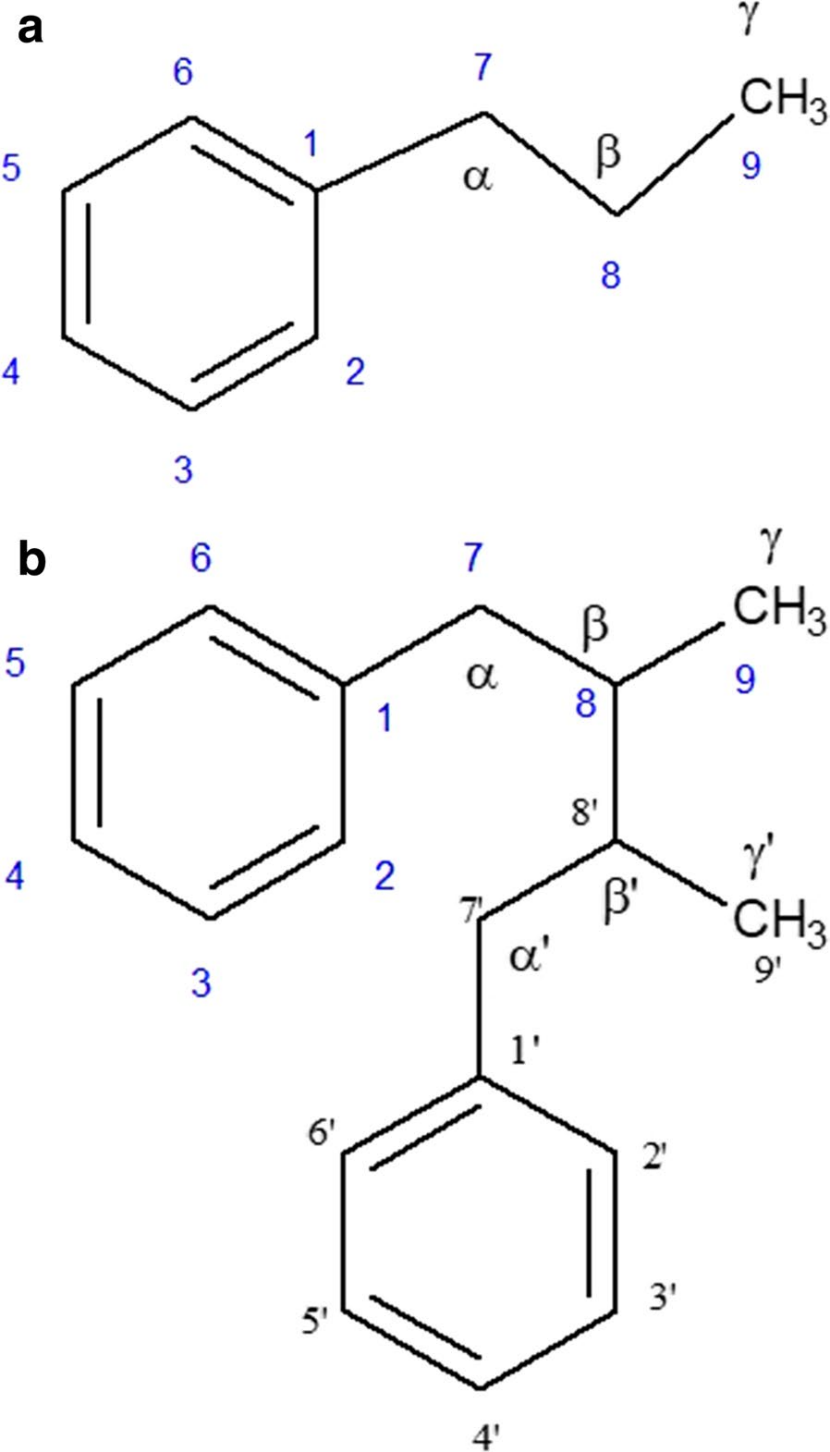
**a** Phenylpropane unit. **b** Dimer of C6-C3 unit via β-β′ bonding

**Fig. 3:**
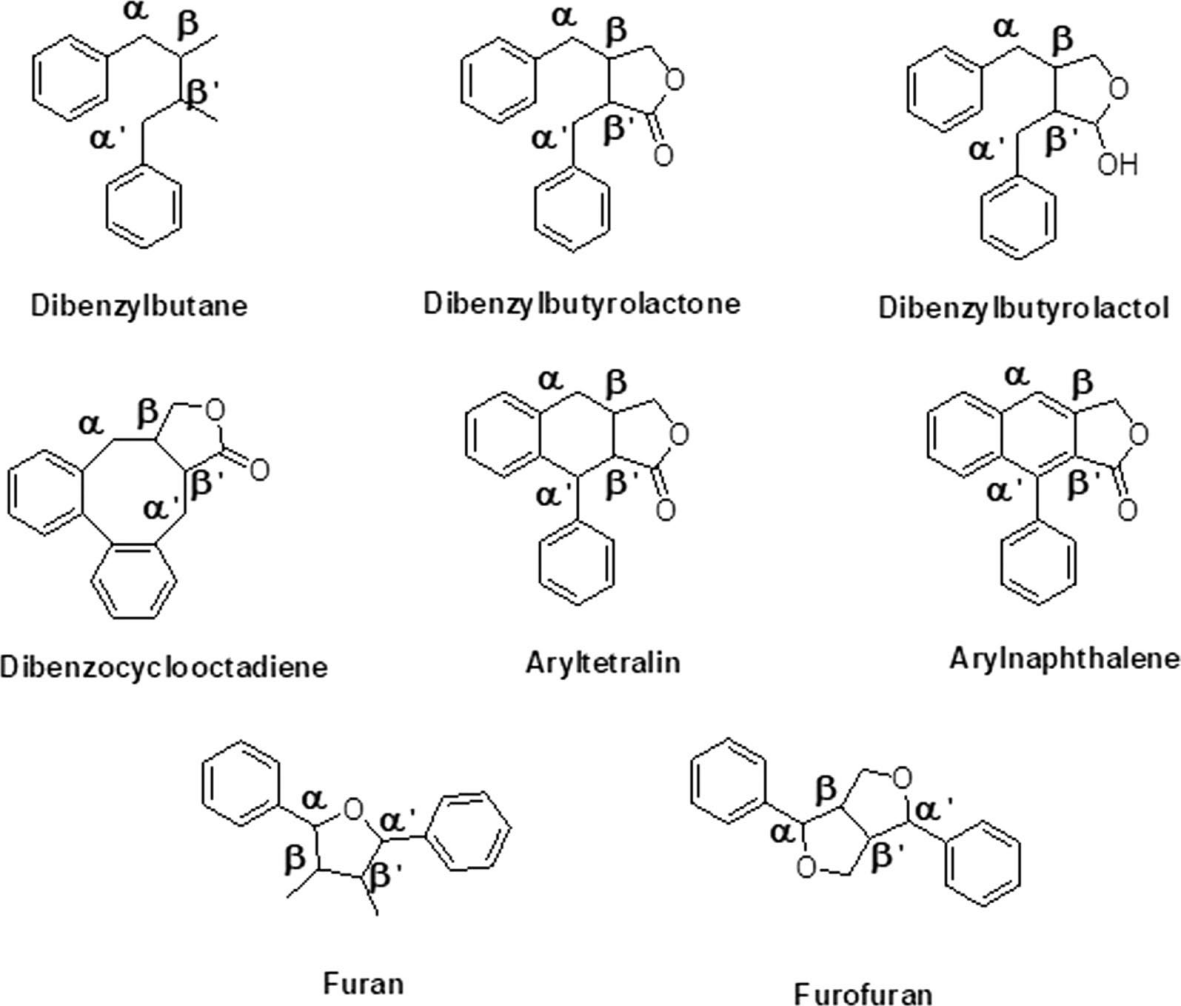
Eight chemical classes of lignan molecules

It is well-established that the supergroup of natural phenolics is biosynthesized through the shikimic acid pathway. The biodiversity of this lignan class of molecules is found in various parts of more than 60 families of plants and they are potential bioactive principles toward cancerous cells. Beside their cytotoxic property they are also useful to treat diabetes, oxidation of living cells as antioxidants, cardiovascular diseases, microbial infections, and other major or minor inflammatory responses [23, 24]. As per the earlier findings, the basic structure of lignan contains the nine carbon (in a C6-C3 fusion) phenylpropane unit (Fig. 2a) from cinnamyl structures [25] which was redefined by Haworth [18] as dimer of C6-C3 unit via β-β′ bonding (Fig. 2b). Besides this basic hydrocarbon skeleton they possess numerous additional side groups either in the form of aliphatic or aromatic origin and they are classified accordingly. There are eight subtypes of major lignans (Fig. 3) such as dibenzylbutane (e.g., Enterodiol), dibenzylbutyrolactone (e.g., Enterolactone), dibenzylbutyrolactol (e.g., Gnetucleistol F), dibenzocyclooctadiene (e.g., Gomisins), aryltetralin (e.g., Podophyllotoxins), arylnaphthalene (e.g., Justcidins), furan (e.g., Belischmins), and furofuran (e.g., Epimagnolin) derivatives. Except these eight subtypes, they are also diversified based on the presence or absence of oxygen [26, 27]. Hybrid lignans are molecules which have other secondary metabolites like flavonoids (flavolignans), coumarins (coumarinolignans), xanthones (xantholignans), stilbenes (stilbenolignans), etc., and possess lignan like biological and chemical properties.

**Fig. 4:**
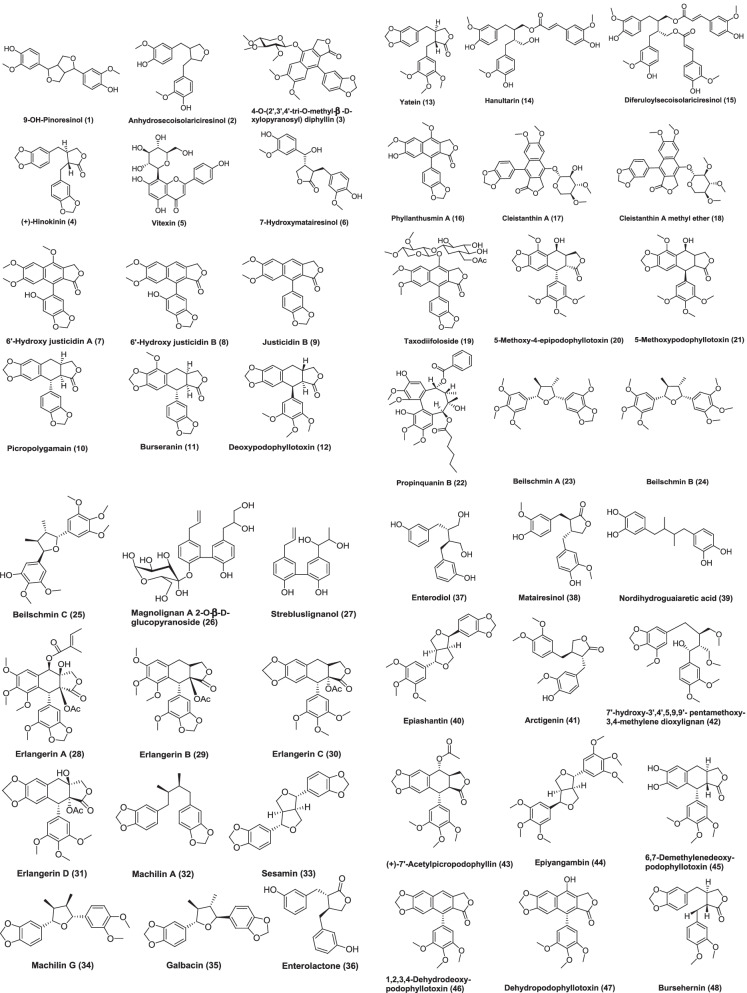

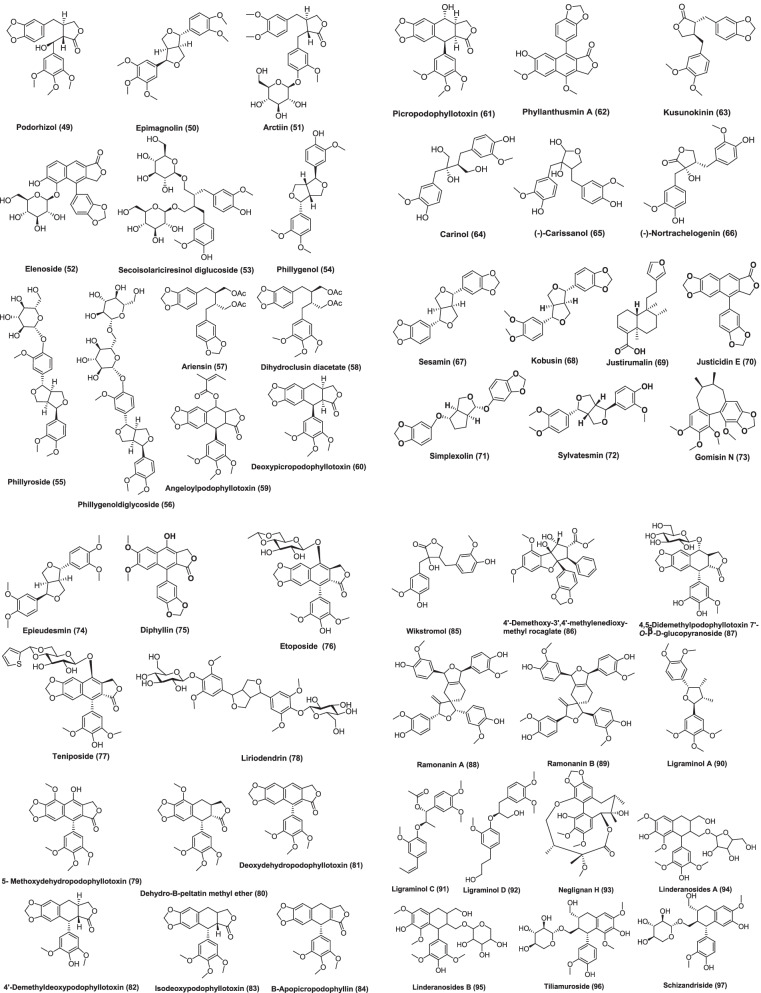

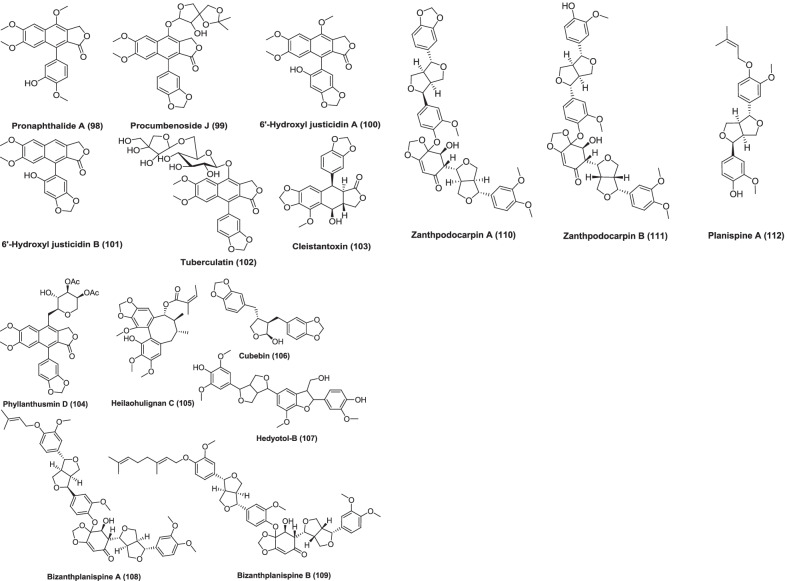
Chemical structures of anticancer isolated lignans from plants

The discovery of Podophyllotoxins as gold standard in leading lignans along with establishing its cytotoxic property and topoisomerase-II inhibitory potentials helped the research community to develop other clinically important drugs like etoposide, teniposide, clinical candidates like Etopophos, NK611, GL331, etc. [28]. Ward reported a total number of 83 synthetic and transformational schemes including stereospecific and asymmetric consideration [29] to obtain them in laboratory.

There is persistent interest in the cancer-protective effects of lignans, which have been shown to have an advantageous anti-tumor effect throughout the early phases of carcinogenesis. The present review, summarizes the recent literature which deals with the lignans isolated from plants having anticancer potential with their reported mechanism of action which are listed in Table 1. Lignans has been considered as the promising anticancer agents.

**Table 1:** List of lignans isolated from plant with anticancer activity

Name	Structure ID (Fig. 4)	Source	Extract	Part	Cancer cell line used (in vitro)	IC_50_	In vivo	Dose	Comments	Reference
9-OH-Pinoresinol	1	*Saussurea salicifolia* (L.) DC, Asteraceae	Chloroform fraction of ethanolic extract	Aerial parts	L5178Y	–	–	10 μg/mL	Ethanolic extract of the plant reduced the growth of leukemia mouse lymphoma cells to 23.8%. It looks like lignan 9-OH-pinoresinol is responsible for the activity which is well known from other plant sources	[30]
Anhydrosecoisolariciresinol	2	*Linum usitatissimum*, Linaceae	–	Seeds	MCF-7			100 µM	The isolated lignan causes 30% inhibition of cell growth as compared to control	[31]
4-O-(2′,3′,4′-tri-O-methyl-β-D-xylopyranosyl) diphyllin	3	*Phyllanthus taxodiifolius*, Phyllanthaceae	–	Aerial parts	HCT116	0.08 ± 0.03 µM			In vitro studies has been shown to inhibit the growth of a number of cancer cell*.* It shows strongest antiproliferative effect on HCT116 cells. The compound induces apoptosis in HCT116 cells by activating caspase-3 pathway and antiproliferative effect is due to promotion of microtubule depolymerization	[32]
( +)-Hinokinin	4	*Wikstroemia lanceolata*, Thymelaeaceae	Methanol	Stems and roots	P-388	1.54 µg/mL (ED_50_)			Showed significant cytotoxic activity	[33]
Vitexin	5	*Vitex negundo*, Verbenaceae	Ethanol	Seeds	MCF-7, ZR-75-1, SK-BR-7, MDA-MB-231, MDA-MB-435s, PC-3, LNCaP and COC1	100 mg/kg	In vivo studies done using tumor xenograft models like MCF-7, MA782, MDA-MB-435s, and T47D xenografts for breast, PC-3 for prostate, HeLa cells for cervical, and HepG2 for liver xenograft		Vitexins (lignan mixture) has cytotoxic effects on MCF-7, ZR-75-1, SK-BR-7, MDA-MB-231, MDA-MB-435s, PC-3, LNCaP COC1 cancer cells. Vitexin induced antitumor effect and cytotoxic activity is exerted through proapoptotic process, which is mediated by a decreased Bcl-2/Bax ratio and activation of caspases	[34]
7-Hydroxymatairesinol (HMR)	6	*Picea abies*, Pinaceae	Acetone–water (9:1)	Heartwood			LNCaP human prostate cancer xenografts in athymic nude male mice		There is significant decrease in tumor volume. A control diet supplemented with 0.15% or 0.30% of HMR was administered to mice and the tumor take rate and growth was observed for 9 weeks. The diet supplemented with HMR has been shown to inhibit the growth of LNCaP tumors. Mice treated with HMR had smaller tumor volume, lower tumor take rate, increased proportion of non-growing tumors, and higher tumor cell apoptotic index compared with controls. Cell proliferation index was also decreased in mice receiving the 0.30% HMR diet when compared with mice receiving the control diet	[35]
6′-Hydroxy justicidin A, 6′-hydroxy justicidin B, justicidin B	7, 8, 9	*Justicia procumbens*, Acanthaceae	Ethanolic	Whole plant	K562	20, 43.9 and 45.4 µM			All the compounds significantly inhibited the growth of K562 cells by decreasing both proliferation and SOD activity and inducing apoptosis in dose-dependent manner. Activation of caspase-3 pathway suggests that these compounds induce apoptosis through caspase intrinsic or extrinsic pathway	[36]
Picropolygamain, Burseranin	10, 11	*Bursera graveolens*, Burseraceae	Methanol	Stem	HT1080	1.9, 5.5 µg/mL (ED_50_)			Showed significant cytotoxic activity	[37]
(-)-Deoxypodophyllotoxin, (-)-yatein	12, 13,	*Hernandia nymphaeifolia*, Hernandiaceae	Methanol	Bark	P-388, KB16, A549, HT-29	< 1 µg/mI (ED_50_)			Showed significant cytotoxic activity	[38]
Hanultarin, 1,4-O-Diferuloylsecoisolariciresinol	14, 15	*Trichosanthes kirilowii*, Cucurbitaceae	80% Aqueous methanol	Seeds	A549, SK-Mel-2, B16F1	3–13 µg/mL			Inhibitory effect on the polymerization of the actin cytoskeleton in normal epidermal keratinocyte (HaCaT cells) has been shown by compound Hanultarin as compared to those of the other isolates	[39]
Phyllanthusmin A	16	*Phyllanthus oligospermus*, Phyllanthaceae	Chloroform fraction of methanolic extract	Stems and roots	KB and P-388	2.24 µg/mL and 0.13 µg/mL			Showed significant cytotoxic activity	[40]
Cleistanthin A, Cleistanthin A methyl ether, Taxodiifoloside	17, 18, 19	*Phyllanthus taxodiifolius*, Euphorbiaceae	Ethanol fraction of methanol extract	Aerial parts	Five cultured mammalian cell lines. P-388, KB, Col-2, MCF-7 and Lu-1	Compounds showed GI_50_ value in the range 10^–6^–10^–9^ M			Cleistanthin A, Cleistanthin A methyl ether has shown potent cytotoxic activity and Taxodiifoloside showed moderate activity	[41]
5-Methoxy-4-epipodophyllotoxin, 5-methoxypodophyllotoxin	20, 21	*Libocedrus chevalier*, Cupressaceae	Ethyl acetate	Bark	KB	45 µM and 11 µM			Both isolated lignans were also evaluated for their tubulin assembly inhibitory activity. 5-methoxy-4-epipodophyllotoxin inhibited the assembly of tubulin into microtubules with an IC_50_ of 9 µM, whereas the IC_50_ of 5-methoxypodophyllotoxin was 5 µM	[42]
Propinquanin B	22	*Schisandra propinqua* (Wall.), Schisandraceae	Chloroform	Stems	HL-60, Hep-G2, R- Hep-G2, KB, Bel-7402	7.15, 9.81, 14.00, 11.70, 18.81 µM			Cell cycle study and Hoechst 33,258 staining assay suggests that cytotoxic activity of compound may be due to induction of apoptosis	[43]
Beilschmin A, Beilschmin B, Beilschmin C	23, 24, 25	*Beilschmiedia tsangii*, Lauraceae	-	Stems	P-388 and HT-29	1.2 and 5.0 µg/mL 2.2 and 5.1 µg/mL 3.6 and 10.5 µg/mL			Showed significant cytotoxic activity	[44]
Magnolignan A 2-O-β-D-glucopyranoside, Strebluslignanol	26, 27	*Streblus asper*, Moraceae	Chloroform fraction of 75% ethanol	Heartwood	Hep-2 and Hep-G2	13.3 μM, 46.4 μM and 10.1 μM, 21.7 μM			Both lignans showed medium cytotoxic activity	[45]
Erlangerin A to D	2, 29, 30, 31	*Commiphora erlangeriana*, Burseraceae	Resin		EAhy926 and HeLa, L929 and RAW 264.7	68 ± 6, 40 ± 5, 90 ± 5 and 44 ± 9 µg/mL (EC_50_) 23 ± 1.4, 4.0 ± 1.4, 68 ± 6 and 28 ± 0.3 (EC_50_) 0.16 ± 0.09, 0.55 ± 0.007, 5.6 ± 1.5 (EC_25_), and 0.97 ± 0.21 µg/mL (EC_50_) 0.026 ± 0.007, 0.026 ± 0.009, 3.5 ± 1 µg/mL(EC_25_), 0.11 ± 0.017 µg/mL (EC_50_)			Erlangerins C and D were similar to podophyllotoxin on the basis of their structure and biological activity so may have same mechanism of action. They induced a concentration-dependent cytotoxicity in RAW 264.7 and cytostatic effect in HeLa, EAhy926, and L929 cells. But Erlangerins A and B suppressed cell viability at relatively higher concentrations when compared with Erlangerin C and D	[46]
Machilin A, (-)-Sesamin, Machilin G, ( +)-Galbacin	32, 33, 34, 35	*Machilus thunbergii*, Lauraceae	Dichloromethane	Bark	HCT-15, MCF-7 and A549	12.4, 12.4 and 7.9 µM 4.4, 3.4 and 11.0 µM 1.4, 2.7 and 8.3 µM 6.2, 7.9 and 7.9 µM			PLCγ1 plays a key role in proliferation and progression of human cancer. These compounds inhibit PLCγ1 and showed strong antiproliferative activity	[47]
Enterolactone, Enterodiol	36, 37	Mammalian lignans			LNCaP	57 mM and 100 mM	10–100 microM		Growth of prostate cancer cells were suppressed may be by hormonally dependent and independent mechanisms	[48]
Matairesinol	38	*Carthamus tinctorius*, Asteraceae	–	Seeds	HL-60	60 μM			DNA content histogram was analyzed by flow cytometry and it showed rapid increase in subdiploid cells and a concomitant decrease in diploid cells exposed to 100 μM matairesinol. It was concluded that cell death was due to the DNA damage and apoptosis	[49]
Nordihydroguaiaretic acid	39	*Larrea tridentata* DC. Coville, Zygophyllaceae	Resinous exudate	Bush	SW480	1.9 ± 0.5 µg			It caused time and dose-dependent loss of mitochondrial membrane potential (MMP), down regulation of the anti-apoptotic protein bcl_xl_ and an increase of the apoptotic index. It also induced a shift of the culture population to the G2/M phase of the cell cycle	[50]
Epiashantin	40	*Artemisia absinthium* L., Asteraceae	–	Warmwood	SW480	9.8 ± 4.5 µM			The compound caused a time and dose-dependent loss of mitochondrial membrane potential (MMP), down regulation of the anti-apoptotic protein bcl_xl_ and an increase of the apoptotic index	[50]
Arctigenin	41	*Arctium lappa* L., Asteraceae	–	Root	SW480	16.5 ± 8.5 µM			The compound caused a time and dose-dependent loss of mitochondrial membrane potential (MMP), down regulation of the anti-apoptotic protein bcl_xl_ and an increase of the apoptotic index	[50]
7′-Hydroxy-3′,4′,5,9,9′-pentamethoxy-3,4-methylene dioxylignan	42	*Phyllanthus urinaria*, Phyllanthaceae	Ethyl acetate	Whole plant	HEp-2	4.46 µM			7′-hydroxy-3′,4′,5,9,9′-pentamethoxy-3,4-methylene dioxylignan was capable of inhibiting telomerase activity and also could inhibit bcl_2_ and activate caspase 3 and caspase 8 whose significance in the induction of apoptosis is well known	[51]
( +)-7′-Acetylpicropodophyllin, Epiyangambin	43, 44	*Hernandia ovigera* L., Hernandiaceae	Ethyl acetate	Twigs	JB6	0.15 and 0.4.2 µg/mL			Significant inhibition of the transformation of murine epidermal JB6 cells,	[52]
Deoxypodophyllotoxin, 6,7 Demethylenedeoxypodophyllotoxin, 1,2,3,4-Dehydrodeoxypodophyllotoxin, Dehydropodophyllotoxin, Bursehernin, Podorhizol, Epimagnolin	12, 45, 46, 47, 48, 49, 50	*Hernandia ovigera* L., Hernandiaceae		Seeds	Epstein-Barr virus early antigen activation (EBV-EA) induced by 12-O tetradecanoylphorbol 13-acetate (TPA) in Raji cells	550 mol ratio/32 pmol TPA, 510 520 470 470 480 590			Inhibitory effects on EBV activation has been shown by all isolated compounds	[53]
Arctiin, Arctigenin	51, 41	*Saussurea medusa*, Composite	Methanol	Aerial parts			Two stage skin carcinogenesis model using DMBA (7,12-dimethylbenz[a]anthracene) and TPA (12-O-tetradecanoyl phorbol-13-acetate)		Both lignans arctiin and arctigenin exhibited a significant inhibitory effect on the tumor promotion induced by DMBA and TPA by both topical application and oral administration. When both compounds were administered orally reduction in papillomas per mouse at 15 weeks of promotion in case of arctigenin was 4.2 ± 0.1 and Arctiin 4.0 ± 0.2, and at 20 weeks of promotion arctigenin was 6.1 ± 0.1 and Arctiin was 6.1 ± 0.2	[54]
Elenoside	52	*Justicia hyssopifolia* L., Acanthaceae	Ethanolic	Leaves	CCRFCEM, K-526, MOLT-4, RPMI-8226	79–97% growth inhibition		10^–4^ M	Elenoside was cytotoxic to leukemic cell lines (CCRFCEM, K-526, MOLT-4, RPMI-8226) at a concentration of 10^–4^ M (79–97% growth inhibition). Elenoside does not show significant activity at concentration less than 10^–4^	[55]
Secoisolariciresinol diglycoside	53	*Linum usitatissimum*, Linaceae	Ethanolic	Seeds			Female Sprague–Dawley rats	2.93 mmoles/g	Increased plasma insulin-like growth factor I (IGF-I) concentrations are associated with increased breast cancer risk. Secoisolariciresinol diglycoside reduced plasma IGF-I levels. It inhibit Mammary tumor development in rats	[56]
Phillygenol Phillyroside Phillygenoldiglycoside	54, 55, 56	*Lancea tibetica*, Mazaceae			SMMC-7721, HeLa, V79, B16				Phillygenol has shown strong cytotoxic activity on the tested cell lines whereas Phillyroside and Phillygenoldiglycoside had little effect on the proliferation of the tested cell lines	[57]
Podophyllotoxins	*45, 46, 47…*	*Podophyllum peltatum, Podophyllum emodi, Podophyllum versipelle, Linum Juniperus*			small-cell lung cancer (SCLC) dose: > 1 µg/mL (etoposide)				Disrupt the organization of the karyokinetic spindle single-strand and double-strand breaks in DNA through their interactions with DNA topoisomerase II induce cell cycle arrest in the G2-phase of the cell cycle	[17]
Ariensin Burseran Dihydroclusin diacetate	57, 11, 58	*Bursera microphylla* A. Gray, Burseraceae	Methanol	Resin obtained from the bark of the plant	RAW264.7, M12.C3.F6 murine cancer cell line (macrophages transformed by virus Abelson leukemia)	9.8, 0.4, 0.2 μM for all three isolated compounds in RAW264.7 and 2.5 μM for Dihydroclusin diacetate in M12.C3.F6			Dihydroclusin diacetate was shown to be active against both murine cancer cell lines while ariensin, burseran, were active against only RAW246.7 murine cell line only	[58]
(-)-Hinokinin	4	*Zanthoxylum pistaciiflorum* Hayata, Rutaceae	Methanol	Stem Bark	HT-29 cell line	3.52 µg/mL (ED_50_ value)			Showed significant cytotoxic activity against HT-29 cell line	[59]
(-)-Deoxypodophyllotoxin, Angeloylpodophyllotoxin, Deoxypicropodophyllotoxin, Picropodophyllotoxin	12, 59, 60, 61	*Anthriscus sylvestris* Hoffm., Umbelliferae	Methanol	Roots	HL-60				Compounds have an apoptosis-inducing effect in HL-60 cells and it was determined by caspase-3 activation and DNA fragmentation. Typical ladders of DNA fragmentation were observed when treated with compound angeloylpodophyllotoxin, picropodophyllotoxin at 1 mM and (-)-Deoxypodophyllotoxin at 0.01 mM	[60]
Phyllanthusmin A	62	*Phyllanthus oligospermus*, Phyllanthaceae	Chloroform fraction of methanol extract	Stems and roots	KB and P-388	2.24 and 0.13 µg/mL			Phyllanthusmin A showed significant cytotoxicity	[61]
(-)-Kusunokinin	63	*Piper nigrum*, Piperaceae	Dichloromethane	Fruits	MCF-7 and MDA-MB-468	1.18 and 1.62 µg/mL			This compound induced cell apoptosis and drove cells toward the G2/M phase which is determined by cell studies. It also decreases topoisomerase II and Bcl-2. There is increase in p53, p21, bax, cytochrome c, and caspase-8, -7, and -3 activities, except caspase-9. This shows that kusunokinin has potent anticancer activity through the extrinsic pathway and G2/M phase arrest	[62]
Yatein	13	*Austrocedrus chilensis*, Cupressaceae	Methanol	Heartwood	P3X63-Ag8.653	Yatein exhibited potent cytotoxicity, inducing 75% cell death at 25 mg/mL after 24 h of treatment			Yatein showed toxicity in P3X cells in a dose-dependently. In cells that survived to yatein treatment, the microtubular apparatus was altered, as determined by immunofluorescence techniques, and SEM and TEM analyses displayed changes in morphological and ultrastructural level. There was alteration in cell shape and membrane system was damaged	[63]
(-)-Carinol, (-)-Carissanol, and (-)-Nortrachelogenin	64, 65, 66	*Carissa spinarum* L., Apocynaceae	Methanol	Stem	MCF7 and A549	< 1 µg/mL 11.0 and 17.4 µg/mL 29.0 and 88.3 µg/mL			The most active lignan was (-)-carinol and (-)-carissanol was more potent than (-)-nortrachelogenin	[64]
Sesamin, Kobusin, 4′O Demethyl magnolin	67, 68	*Zanthoxylum alatum*, Rutaceae	Petroleum ether	Stem bark	A549 and MIA-PaCa	37.46 ± 1.097 and 34.04 ± 1.7621 34.71 ± 2.331 and 32.86 ± 2.0271 26.47 ± 1.871 and 26.47 ± 1.871 mg/mL			Cytotoxic activity has been shown by all three isolated lignans in different ranges. 4′O dimethyl magnolin was the novel bioactive compound from a plant source and found to be most active. In apoptosis study, treatment caused typical apoptotic morphological changes. It enhances the apoptosis at IC_50_ dose (21.72 mg/mL) on MIA-PaCa cell line. This compound induce apoptosis as the mechanism of cell death	[65]
Justirumalin	69	*Justicia neesii*, Acanthaceae			MCF-7, AGS	42.8 and 42.1%, inhibition, respectively		25 μg/mL	Justirumalin inhibited human stomach and breast cancer cells	[66]
Justicidin E, Simplexolin	70	*Justi caorbiculata*, Acanthaceae			MCF-7, SF-268, CNS, NCI-H460, HCT-116 and AGS			25 μg/mL	Justicidin E inhibited the proliferation of lung, breast and colon cancer cell lines with inhibition values ranged between 40 and 53% and simplexolin gave 40–50% inhibition against lung, breast, colon, and CNS cancer cell lines when tested at 25 µg/mL	[66]
Sylvatesmin	72	*Lancea tibetica* Hook. f. et Thoms, Scrophulariaceae	Methanol	Whole plant	B16, SMMC-7721, Hela	40.4 ± 1.4 mg/mL, 113.4 ± 2.16 mg/mL, 127.9 ± 3.20 mg/mL		25 µg/mL	Sylvatesmin exhibited the effective antitumor activity, especially on B16 cells	[67]
Gomisin N	73	*Schisandra chinensis* (Turcz.) Baill., Schisandraceae or Magnoliaceae	Dichloromethane	Ripe berries	HT-29	43 µM			Effective against colorectal proliferative processes	[68]
Epieudesmin	74	*Hernandia nymphaeifolia* (Presl) Kubitzki, Hernandiaceae	CH_3_OH/CH_2_Cl_2_ (1:1) extract	Fruits	A549, MCF-7 and HER2, MDA-MB-231	5.7 µM, 8.1 µM, 231 8.2 µM			Compounds displayed significant anti proliferative activity	[69]
Podophyllotoxins, Diphyllin, Etoposide (VP–16), teniposide	12, 75, 76, 77	*Podophyllum peltatum,* Berberidaceae		Whole plant	P-388, HT-29, A-549 and MEL-28				This bioactive lignan is very effective on small cell lung cancer, malignant lymphoma, and testicular carcinoma It is also potent on Wilms tumors, ovarian cancer, brain tumors, urinary tract cancer, etc.	[70]
Liriodendrin	78	*Plumeria rubra*, Apocynaceae	Water soluble fraction of methanolic extract	Stem bark	P-388 murine lymphocytic leukemia and human cancer cell types (fibrosarcoma, melanoma, breast, lung, colon and KB)	P-388—2.4 µg/mL Fibrosarcoma—98.9 µg/mL Melanoma—19 µg/mL Breast cancer—30 µg/mL Lung cancer—6.0 µg/mL Colon cancer—16 µg/mL KB—6.0 µg/mL (ED_50_ values)			Exhibit cytotoxic activity	[71]
5- Methoxydehydro podophyllotoxin, dehydro-β-peltatin methyl ether, Dehydropodophyllotoxin, Deoxydehydropodophyllotoxin, Yatein, 4′-Demethyldeoxypodophyllotoxin, Isodeoxypodophyllotoxin, Deoxypicropodophyllin, β-apopicropodophyllin	79, 80, 47, 81, 13, 82, 83, 60, 84	*Hyptis verticillata,* Lamiaceae	Chloroform	Aerial parts	P-388, HT-low, KB, A43l, ZR-75-1, LNCaP and U373	4.0, 15.6, 6.0, 6.2, > 20, 11.6 and 16.3 µg/mL 1.8, 3.4, 2.2, > 20, > 20, 3.2, and 5.9 µg/mL > 5, 9.7, 5.0, > 20, > 20, 11.7 and > 20 µg/mL > 5, > 20, 11.4, 6.2, > 20, 11.6 and > 20 µg/mL 0.4, 0.07, 0.08, > 20, 0.5, 0.16, and 0.3 µg/mL 0.005, 0.01, 0.01, 0.08, 2.1, 0.02 and 0.1 µg/mL > 20, 10.7, 6.7, 6.2, 13.2, 12.0 and 2.9 µg/mL 0.1, 0.2, 0.1, > 20, 0.6, 0.2 and 0.1 µg/mL 0.002, 0.003, 0.05, 4.3, 2.0, 0.01 and 0.001 µg/mL. (ED_50_ values)				[72]
Wikstromol	85	*Wikstroemia foetida var.oahuensis* and *Wikslroemia uwa-ursi* Gray Thymelaeaceae	Chloroform fraction of ethanolic extract	Whole plant			P-388 Iympho cyticleukemia (3PS) test system	16, 10, 4, 2, and 1 mg/kg	Wikstromol demonstrate activities of 154, 146, 137, 141, and 130% test/control at dose of 16, 10, 4, 2, and 1 mg/kg, respectively	[73]
4′-Demethoxy-3′,4′-methylenedioxy-methyl rocaglate	86	*Aglaia elliptica* Bl., Meliaceae	Chloroform	Stem	HT-1080, KB, A431, LNCaP, ZR-75-1, and U373, BCl	10.0, 6.0, 10.0, 2.0, 2.0, 0.8, 0.9 ng/mL	Antitumor potential of compound was performed with female Balb/c athymic nude mice. Compound significantly inhibited the growth of BC1 cells in culture. The growth of tumor was retarded by treatment with isolated compound during the first 23 days of the study, but after that tumor growth paralleled to the control group		This compound acts by cytostatic mechanism, rather than inducing necrosis or apoptosis. Cells were transiently blocked in the G1/G0 phases of the cycle, and this may be due to inhibition of protein biosynthesis	[74]
4,5-Didemethylpodophyllotoxin 7′-O-b-D-glucopyranoside	87	*Sinopodophyllum emodi*, Berberidaceae	n-butanol	Roots and rhizomes	Hela, K562, SH-SY5Y and CNE				Compound showed cytotoxicity against four human cancer cell lines	[75]
Ramonanin A Ramonanin B	88, 89	*Guaiacum officinale*, Zygophyllaceae	Chloroform	Heartwood	MD-MBA 231	18 μM	The ramonanins exhibit cytotoxic activity against human breast cancer cell lines with an IC_50_ value of 18 μM and induce cell death via apoptotic mechanisms		Ramonanin A-treated MD-MBA 231 cells showed characteristic features of apoptotic cell death, which appeared in a time and dose-dependent manner and cell cycle distribution was monitored via flow cytometry using fluorescence-activated cell sorting. It was noted that the ramonanins strongly disrupt cell cycle progression at the G1/S phase transition	[76]
Ligraminol A, Ligraminol C, Ligraminol D	90, 91, 92	*Acorus gramineus*, Araceae	Methanol	Rhizomes	A549, SK-OV-3, SK-MEL-2	6.92, 9.44, and 4.53 μM			Compounds showed weak inhibitory activity against various cancerous cell lines. Study has also been performed to check whether the cytotoxicity was selective between tumor and normal cells. For this compounds were evaluated for normal human cell line, HUVEC. This was noted that cytotoxicity of isolated compounds was higher against tumor cells than normal cells. Ligraminol A showed the highest selective cytotoxicity against the SK-MEL-2 cell	[77]
Neglignan H	93	*Schisandra neglecta*, Schisandraceae	Ethyl acetate layer of 70% aqueous acetone	Stem	NB4, A549 and MCF7	8.1, 7.4 and 6.7 µM				[78]
Linderanosides A and B	94, 95	*Lindera glauca*, Lauraceae	Methanolic	Twigs	A549	20.86 ± 0.94, 21.85 ± 0.61 µM				[79]
Tiliamuroside, Schizandriside	96, 97	*Tilia amurensis* Rupr., Tiliaceae	Methanolic	Trunk	A549, SK-OV-3, SK-MEL-2, and HCT-15	7.32, 8.89, 7.84, and 6.18 μM 6.90, 5.88, 3.26, and 6.65 μM			cytotoxic activity of compounds against the tested cell lines were due to absence of a methoxy group at C-3 in the aryl-tetralin type lignan as indicated by the results	[80]
Pronaphthalide A, Procumbenoside J, 6′-hydroxyl justicidin A, 6′-hydroxyl justicidin B, Tuberculatin	98, 99, 100, 101, 102	*Justica procumbens*, Acanthaceae	Ethanol	Whole plants	Human LoVo and BGC-823	0.03–10.0 μM,				[81]
Cleistantoxin	103	*Cleistanthus indochinensis*, Euphorbiaceae	Dichloromethane	Fruits	KB, MCF-7, MCF-7R	0.022, 0.036, 0.014 μM			Cleistantoxin had strong activity against KB cells also showed significant activity against MCF-7 and MCF-7R	[82]
Phyllanthusmin D	104	*Phyllanthus poilanei*, Phyllanthaceae	Chloroform fraction of methanol extract	Air-dried leaves, twigs, flowers, and fruits	HT-29	170 nM	Compound showed activity when tested in an in vivo hollow fiber assay using HT-29 cells implanted in immunodeficient NCr nu/nu mice	5 μM	Cytotoxic effects of phyllanthusmin D were by inducing tumor cell apoptosis through activation of caspase-3. DNA topoisomerase IIα activity was not inhibited Treatment of HT-29 cells with phyllanthusmin D for 72 h resulted in 28.2% or 30.3% of HT-29 cells undergoing early apoptosis, respectively,	[83]
Heilaohulignan C	105	*Kadsura coccinea*, Schisandraceae	80% ethanol	Roots	HepG-2, BGC-823 and HCT-116	9.92, 16.75 and16.59 µM			heilaohulignan C showed good cytotoxicity in HepG-2 cancer cells and weak cytotoxicity against BGC-823 and HCT-116 cancer cells	[84]
(-)-Cubebin	106	*Piper cubeba*, Piperaceae	Acetone	Seeds	A549, K562, SiHa, KB	8.30 ± 0.16, 8.66 ± 0.43, 8.16 ± 0.41 µM				[85]
Hedyotol-B	107	*Herpetospermum pedunculosum*, Cucurbitaceae	Ethyl acetate	Stems	SGC7901, A549	1.7 ± 0.1 and 6.1 ± 0.5 μM			Hedyotol-B displayed potent inhibitory effect against gastric and lung carcinoma	[86]
Bizanthplanispine A and B, Zanthpodocarpin A and B, Planispine A	108, 109, 110, 111, 112	*Zanthoxylum planispinum* Sieb., Rutaceae	95% aqueous MeOH	Roots	Hela, HL-60, PC-3	Bizanthplanispine A and B, zanthpodocarpin A and B showed significant reduction in the proliferation of Hela with IC_50_ values ranging from 15.00 to 26.44 µg/mL. Planispine A showed the strongest inhibition on the growth of HL-60 and PC-3 with IC_50_ values of 4.90 and 23.45 µg/mL			All isolated compounds showed inhibitory effect on different cancer cell lines	[87]

L5178Y, leukemia mouse lymphoma cells; MCF-7, breast cancer cell lines; HCT116, human colon carcinoma cell lines; P-388, leukemia cancer cells; SK-BR-7, breast cancer cells; MDA-MB-231, breast cancer cells; MDA-MB-435s, breast cancer cells; LNCaP, prostate cancer cells; COC1, ovarian cancer cells; K562, human chronic myeloid leukemia; HT1080, human fibrosarcoma cells; KB16, human epidermoid carcinoma cells; HT-29 human colorectal adenocarcinoma cell line; A549, human lung cancer cell line; SK-Mel-2, human skin melanoma cell lines; B16F1, mouse melanoma cell lines; Col-2, human colon cancer cell lines; Lu-1, lung adenocarcinoma cell line; HL-60, human acute promyelocytic leukemia cell line; Hep-G2, human hepatocellular carcinoma; R-Hep-G2, human resistant hepatoma; Bel-7402, hepatocellular carcinoma; Hep-2 alveolar epithelial carcinoma cell line; EAhy926, human umbilical vein cell line; HeLa, human uterine cervix carcinoma cell lines; L929, murine fibroblast cell line; RAW 264.7, murine macrophage cell line; HCT-15, human colorectal carcinoma cell line; SW480, colon carcinoma cells; JB6, murine epidermal cells; CCRFCEM, leukemia cell lines; K-526, leukemia cell lines; MOLT-4, leukemia cell lines; RPMI-8226, leukemia cell lines; SMMC-7721, human hepatoma cell line; V79, hamster lung fibroblast cell; B16, mouse melanoma cell; M12.C3.F6, murine cancer cell line (macrophages transformed by virus Abelson leukemia); MDA-MB-468, breast cancer cell lines; P3X63-Ag8.653, murine myeloma cell line; AGS, gastric cancer cell lines; MIA-PaCa, pancreatic carcinoma cell line; SMMC-7721, human hepatoma cells; HER2, negative breast cancer cell line; MDA-MB-231, triple negative breast cancer cell line; MEL-28, melanoma cell lines; HT-low, Human fibrosarcoma cells; KB, human oral epidermoid carcinoma; A43l, human epidermoid carcinoma; ZR-75–1, human hormone-dependent breast cancer; U373, human glioblastoma cell lines; BCl, human breast cancer; SH-SY5Y, neuroblastoma cell line; CNE, nasopharyngeal carcinoma; SK-OV-3, ovary malignant ascites; NB4, human acute promyelocytic leukemia cell line; MCF-7R, human breast cancer cell line; SiHa, human cervical carcinoma; SGC7901, human gastric carcinoma; PC-3, human prostate carcinoma cells; SCLC, small-cell lung cancer

## Material and methods

The bibliography was crucially analyzed from worldwide established scientific databases like SCOPUS, PubMed, ScienceDirect, Springerlink, Web of Science, Wiley, SciFinder, and Google Scholar. The botanical names of these selected plant species were verified from the plant list. The inclusion criteria for the selection of data are lignans isolated from Medicinal plants with reported anticancer activity. Both the reviews and the research articles on medicinal plants are considered. The search terms were lignans, anticancer plants containing lignans, chemistry of lignans without narrowing or limiting search items.

## Conclusions

Lignans are secondary metabolites are also phenolic in nature and have diversity in biological activities. Previous studies revealed that the most remarkable bioactivity of lignan class of molecules are antioxidant and anticancer. This review covers a considerable number of naturally obtained lignans that are reported to have anticancer potential. In this review we have tried to sum up the isolated anti-cancerous lignan, its source, active plant part, extract and various cell lines used to establish different studies. Here we have included a total 113 numbers of natural lignans. Many studies that mainly performed in human cell lines have reported inhibition of enzymes that retards tumor growth. Very few plants have been evaluated for their in vivo anticancer activity.

It can be concluded that in near future the lignans may be an effective pharmacon for the treatment of cancer. Fruitful areas of future research may be in modifying natural lignans or synthesizing new lignans with structural diversity and potent pharmacological activities. However, among the vast numbers of existing plants on this planet, only a few species have been studied so far for their anticancer principles. Extensive studies are needed to be done highlighting the mechanism of anticancer action of explored and unexplored plants.

Potent anticancer lignans reported in this review needed to be further explored in clinical trials on different models for their effectiveness, toxicological studies, and also targeting particular genotoxic profile against a wide range of cancer in both *in vitro* and in vivo. These compounds are obtained from plants in very minute quantities so this is one of the main challenges to be addressed in the future and their total synthesis in order to allow further bioactivity studies. The data will definitely attract many researchers to start further experimentation that might lead to the drugs for the cancer treatment and to manufacture new herbal drugs which have significant anticancer potential.

## Data Availability

Not applicable.
